# Psychological Impact of the Very Early Beginning of the COVID-19 Outbreak in Healthcare Workers: A Bayesian Study on the Italian and Swiss Perspectives

**DOI:** 10.3389/fpubh.2022.768036

**Published:** 2022-03-24

**Authors:** Sara Uccella, Francesco Mongelli, Pietro Majno-Hurst, Luca Jacopo Pavan, Stefano Uccella, Cesare Zoia, Laura Uccella

**Affiliations:** ^1^Department of Neurosciences, Rehabilitation, Ophthalmology, Genetics and Maternal and Child Health, University of Genoa, Genoa, Italy; ^2^Child Neuropsychiatry Unit, Istituto Giannina Gaslini, Genoa, Italy; ^3^Neonatology Unit, Istituto Giannina Gaslini, Genoa, Italy; ^4^Surgery and Emergency Department, Ospedale Regionale di Lugano, Lugano, Switzerland; ^5^Diagnostic and Interventional Radiology Department, Centre Hospitalier Universitaire de Nice, Nice, France; ^6^Department of Obstetrics and Gynecology, University of Verona, Verona, Italy; ^7^Department of Obstetrics and Gynaecology, Azienda Ospedaliera Universitaria Integrata (AOUI) Verona, Verona, Italy; ^8^Neurosurgery Department, Fondazione IRCCS Policlinico San Matteo, Pavia, Italy

**Keywords:** COVID-19, early outbreaks, stress, healthcare workers, mental health, hospital management, trauma

## Abstract

**Background:**

We investigated the COVID19-related psychological impact on healthcare workers in Italy and in Italian-speaking regions of Switzerland, three weeks after its outbreak. All professional groups of public hospitals in Italy and Switzerland were asked to complete a 38 questions online survey investigating demographic, marital and working status, presence of stress symptoms and need for psychological support.

**Results:**

Within 38 h a total of 3,038 responses were collected. The subgroup analysis identified specific categories at risk according to age, type of work and region of origin. Critical care workers, in particular females, reported an increased number of working hours, decline in confidence in the future, presence of stress symptoms and need for psychological support. Respondents reporting stress symptoms and those with children declared a higher need for psychological support.

**Conclusions:**

The large number of participants in such a short time indicates for a high interest on topic among health-care workers. The COVID19 outbreak has been experienced as a repeated trauma for many health-care professionals, especially among female nurses' categories. Early evidence of the need of implementating short and long-term measures to mitigate impact of the emotional burden of COVID-19 pandemic are still relevant.

## Background

The Coronavirus 2019 disease (COVID-19) has been a mass casualty event (1), being stressful and intrusive by affecting daily life and putting adaptive skills to the test.

Psychologically, during the early stages of the outbreak, it has been experienced the so-called alarm stage, because of a virus that confined all of us at home (or at work, in the case of health personnel), putting social relationships, freedom, and economy at strain. Feelings of inadequacy or insufficiency are common, and one can experience a sense of being lost and confused (2). Moreover, mass media provide an incessant heavy load of information about COVID-19, that may act as a potential adding stressor to one's experience of the ongoing pandemic (3).

After the initial stun due to the unimaginable experience of the start of the COVID-19 outbreak, anxiety, irritability, and restlessness come forth. Some physical reactions can mimic the COVID-19 but are linked to somatization of stress: palpitations or difficulty in breathing (expression of sympathetic system activation), cognitive reactions like disorientation, sluggish cognitive tempo, anger, sadness and sleep problems may have appeared, as also the Diagnostic Manual of Mental Disorders suggests in relationship to Acute Stress Disorders 5th edition (DSM-5) (4–8).

It is likely that the whole population would have experienced marked stress reactions, especially on subjects suffering from psychological fragilities already before the COVID-19 outbreak (9, 10).

In this sense, attention on healthcare workers (especially doctors and nurses) has been high since the beginning (11), as they have been the first ones (and maybe the most) under both physical and psychological pressure, because of their frontline role and their major risk of becoming infected (2, 5, 11–13). However, a few studies had focused on healthcare team members other than medical professionals (eg. therapists, obstetricians, pharmacists, technical operators including ones working in the canteens, laundry etc., and others) who also had also been involved into the frontlines since the early start of the COVID-19 pandemic (11–13).

For these reasons, an immediate special task force of Italian-speaking medical specialists in the field of either mental health or emergency medicine was created to give a quick qualitative overview of the mental health status of the whole category of healthcare professionals.

The “Coping with COVID-19” (CwCOVID-19) project was developed in early March, during the first days of the COVID-19 outbreak in Italy and Swiss. The purpose of this project was to support healthcare professionals during the first critical phase of the disease management. This was conducted through a dedicated web service managed by specialists in mental health and emergency medicine and specifically dedicated to healthcare workers, available on social networks, which provided information about acute stress.

Furthermore, the project also aimed to conduct an exploratory overview of stress behaviors and coping skills of healthcare professionals during early phases of COVID-19 outbreak management among Italian-speaking regions, such as northern Italy, where the European outbreak began, and southern Switzerland, that followed a few days later.

## Methods

### Study Design and Participants

Cw-COVID-19 is an open project developed in the very early days of COVID-19 outbreak by experienced medical specialists, expert in the field of either mental health or emergency medicine.

Between 14th and 16th March 2020, hospital workers of different public hospitals in Switzerland and Italy were asked to complete an online survey regarding the early psychological impact of the COVID-19 outbreak on daily life, at the time point when the virus mainly widespread in northern Italy, Iran and China, but hadn't assumed extensive dissemination in Switzerland yet. Participants were invited to answer the questionnaire via social media (either Facebook^®^, Instagram^®^, or WhatsApp^®^) and agreed voluntarily to the questionnaire being fulfilled. Voluntary participation was stimulated through the social networks (public posts visible on social networks' profiles of the authors or on open channels and groups, and by word of mouth) to collect data from a large and motivated sample.

All data were anonymized, so that, according to the European and Swiss legislation, no informed consent or institutional review board approval was needed. The study was conducted according with the Declaration of Helsinki.

All subjects' categories working in the hospital teams were included, such as physicians, nurses, therapists, obstetricians, pharmacists, technical operators including ones working in the canteens, laundry etc.. and others.

### The Coping With COVID-19 (CwCOVID-19) Questionnaire

The Coping with COVID-19 (CwCOVID-19) questionnaire (see Supplementary Material available online) was built on Google Forms (https://www.google.com/forms/about/).

It consisted of 38 items, investigating demographic, social, and working status (gender, age, civil status, the total number of households and of family members aged >65-year-old, years worked in the hospital, type of profession, department, changes in working schedules since the beginning of the outbreak. A few items on previous medical or psychological fragilities have been also explored, such as presence of chronic diseases, alcohol drinking, drugs consumption, smoking status and number of cigarettes per-day, social media use, history of anxiety and medical therapy). Moreover, positive coping attitudes have also been asked (such as time spent doing hobbies or physical activity). Inspiring to the DSM-5, acute stress symptoms have been investigated (eating behavior changes, new symptoms occurring in the last three weeks, number of hours spent to get information and to speak about COVID-19, detachment from family due to the confinement, stress behaviors in children, change in physical contacts). Moreover, need for psychological support (actual perception of the need for qualified psychological support and overall trust in the future) was asked. We decided to not use previously validated scales to catch behaviors and psychological changes associated with the peculiar emergency situation, as explorative surveys have been a method already adopted for other research about pandemics (9–11).

The (closed) form is available at the following link:

https://docs.google.com/forms/d/e/1FAIpQLScFrdO8O4zay5-oX41YWsBcI7L1v5nv5YdIAA2qDt5CWVeyJA/closedform.

### Statistical Methods

All anonymized data were collected on an Excel database (Microsoft Excel^®^ 2019) and analyzed.

The exploratory analysis was performed taking into consideration the non-probability nature of the sample, based on unrestricted, self-selected survey, as indicated by Fricker (14).

Statistical analysis was performed using the open-source packages “Pandas,” “NumPy,” “SciPy,” “Seaborn,” and “PyMC” for Mac Os X versions 0.23.0, 11.1.3, 1.1.0, 0.8.1, and 2.3.6, respectively.

Categorical variables are represented either as numbers and percentages in brackets or as medians with interquartile ranges. Continuous variables are listed with means and standard deviations. The Shapiro–Wilk test was performed to evaluate the distribution of the variables.

Our analysis was based on highly Confidence Intervals (CI) of parameter estimates (^*^). Statistical significance was considered achieved whenever CI would be non-overlapping. To compare proportions in different sub-populations, we performed a Bayesian estimation of the parameter distribution for a Bernoulli stochastic variable using a non-informative uniform prior. The posterior was then plotted to have a graphical overview, and credible intervals were computed by assessing the highest density interval (HDI) at 95%. Only as a reference, whenever two proportions had to be compared, we used also a two-tail P test computation based on classic proportion comparison using chi-square. Statistical significance was considered *p* < 0.05. All MCMC runs were checked for adequacy based on Raftery-Lewis diagnostics and by visually inspecting Z score plots. MCMC runs were for 40,000 iterations with a 5,000-iteration burn-in (15). Reasonably low effect size of a sample size greater than 2,500 was considered adequate (16).

## Results

From 14th March 2020, 8.52 p.m. to 16th March 2020, 10.45 a.m., over 38 h, 3,038 hospital workers completed the survey. The percentage of completed surveys was 97.3% as 79 subjects did not answer one or more questions. All the variables had a skewed distribution.

### Sample Characteristics

**Figure 1:**
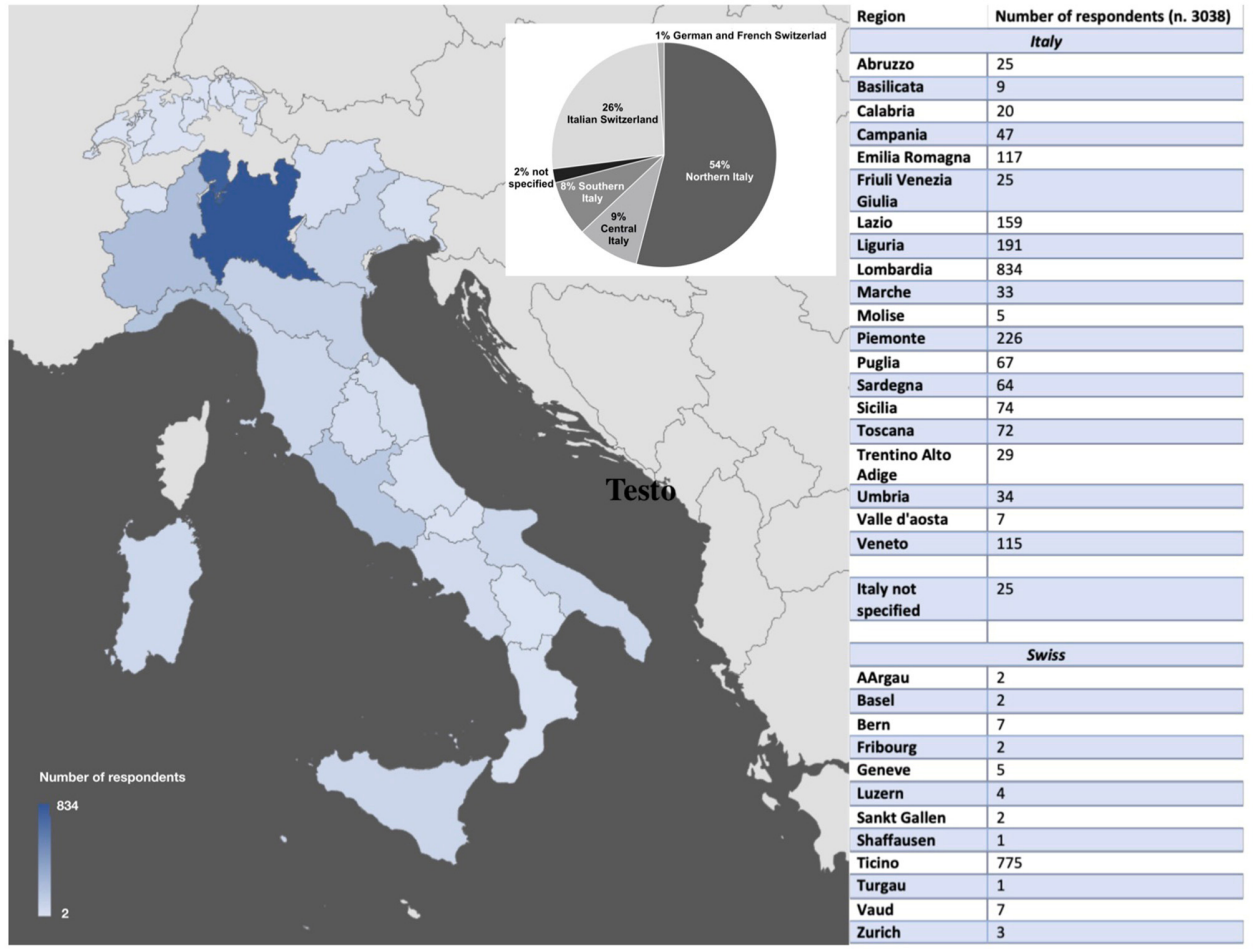
Participants' regions.

Most of our respondents were physicians, female, aged 26–36. Details on age, gender, regions of origin, and working characteristics are reported in Table 1 and Figure 1.

**Table 1:** Demographic, social and work characteristics.

		***N***	**%**
Country	Italy	2,176	71.6
	Switzerland	804	26.4
	Not specified	58	2.0
Sex	Female	2,229	73.7
	Male	795	26.3
	Not specified	14	0.4
Age (year-old)	18–25	114	3.8
	26–35	1,035	34.1
	36–45	996	32.8
	46–55	617	20.3
	56–65	253	8.3
	Over 65	23	0.8
	Not specified	0	0
Kind of job in hospital	Physicians	1,600	53.3
	Nurses	550	18.3
	Radiology, intermediate care technicians	441	14.7
	Ambulance services	87	2.9
	Obstetricians	60	2.0
	Administrative	49	1.6
	Technical services	30	1.0
	Hospitality services	13	0.4
	Pharmacy	7	0.2
	Other hospital services	167	5.6
Civil status	Married	1,313	43.3
	Single	837	27.6
	Stable relationship	672	22.2
	Divorced	172	5.7
	Widowed	25	0.8
	Civil Partnership	13	0.4
	Not specified	6	0.1
Family (number of	1	615	20.3
members)	2	769	25.4
	3	700	23.1
	4	663	21.9
	5	184	6.1
	>5	97	3.2
Children <18-year-old	0	1,734	57.3
in household	1 or more	1,304	42.7
Elderly >65-year-old in	0	2,422	80.0
household	1 or more	616	20.0
Years of work in	0–1 years	207	6.8
hospital	2–5 years	623	20.5
	5–0 years	634	20.9
	10–20 years	858	28.3
	20–30 years	478	15.8
	>30 years	233	7.6
	Not specified	7	0.2
Physical activity	Once a day	180	5.9
	4-5 times a week	185	6.1
	2-3 times a week	855	20.3
	Once a week	439	14.5
	Less than once a week	295	9.7
	None at all	1,081	35.6
Increased number of	No	2,110	70.0
working hours	Up to 10 h	673	22.3
	Up to 20 h	161	5.3
	Up to 30 h	40	1.3
	>30 h	30	1.0

*Characteristics are expressed as absolute value and percentage*.

Seventy percent of respondents declared not to have any additional work due to the COVID-19 emergency. Among respondents who answered to have to work more, 59 were ambulance personnel, 38 nurses, 36% administrative personnel, and 30% physicians. Dividing for department, 60 of workers in the intensive care units, 50 of anesthesiologists, 50 of the staff in the emergency departments, and 46% of general practitioners declared to work more than normal.

### Risk Categories Among Healthcare Workers, Based on Working Experience

Respondents working in hospitals in the last 20–30 years had the highest percentage of increased number of working hours. By grouping working categories in critical care (intensive care unit, emergency department, anesthesiology, ambulance service), surgery (otorhinolaryngology, neurosurgery, orthopedics, cardiac surgery, urology, gynecology), and general practitioners, surgery resulted to be the group that had to reduce the most the number of working hours (C.I. 25.0–31.7%), whereas the other two groups had to work more (C.I 44.7–52.3% for critical care and 40.8–54.8% for general practitioners). On the contrary, 2,256 (76.4%) subjects did not have to reduce their working hours because of COVID-19 emergency, while 308 (10.4%) had to reduce the work up to ten hours per week, 92 (3.1%) up to 20 h per week, 121 (4.1%) up to 30 h per week. One-hundred and fifty (5.1%) respondents had to be quarantined and 24 (0.8%) reported getting infected with the virus. Twenty-three percent of physicians declared to have to work less than normal. As expected, respondents working in southern Italy (C.I. 16.6–22.8%), where the COVID-19 did not spread yet, declared to work less than their counterparts in northern Italy (C.I. 31.1–35.6%) and Switzerland (C.I. 33.5–48.4%).

### Pre-existing Risk Factors for Developing Stress Symptoms Among Healthcare Workers

Almost a half of our sample (1,538 respondents, 50.6%) declared to have a chronic health problem, of whom roughly a quarter (348 subjects, 22.6%) experienced a worsening in their chronic health condition. Appearing of new symptoms were reported by 969 individuals (31.9%) and, more specifically, 285 (9.4%) experienced palpitations, 276 (9.1%) respiratory symptoms, 124 (4.1%) pain, and 285 (9.4%) other symptoms.

### Stress Symptoms

Details are reported in Table 2. problems were the category at higher risk to suffer from sleep disturbances (accuracy 82.9%).

**Table 2:** Stress symptoms.

		***N***	**%**
Sleeping	Sleep less than usual	1,203	39.6
	Sleep the same number of hours but they feel less rested	953	31.4
	No change	678	22.3
	Sleep more than usual	172	5.7
	Sleep the same number of hours but they feel more rested	30	1.0
Eating	No change	1,164	38.4
	More than usual	715	23.6
	Less than usual	529	17.4
	Same quantity of food, but healthier	223	7.3
	Same quantity but more unhealthy food	402	13.3
Smoking	Never smoked	1,820	59.9
	Started before the last three weeks	652	21.5
	Quitted before the last three weeks	487	16.1
	Started in the last three weeks	27	0.9
	Quitted smoking in the last three weeks	41	1.4

*Characteristics are expressed as absolute value and percentage*.

Eating habits changed in 1,869 (61.5%) hospital workers and 679 (22.3%) declared to be active smokers. Among them, 268 subjects (39.5%) had to increase the number of cigarettes (cigars, e-cigarettes, etc.) smoked per day in the last 3 weeks. One-thousand-six-hundred-fifty-seven respondents (55.1%) stated to drink alcohol occasionally or regularly. Among them, 57 (3.4%) started in the last 3 weeks and 255 (15.3%) declared that their consumption had increased in the last 3 weeks. The vast majority of respondents said that they never used drugs (2,756, 91.1%), whereas 129 quitted before the last 3 weeks (4.2%) and three (0.1%) within the last 3 weeks.

Regarding the item investigating time spent on searching information about COVID-19, 654 respondents (21.1%) declared less than an hour per day, 1112 (36.7%) between one and 2 h, 1,094 (36.1%) more than 2 h per day, and 173 (5.7%) did not look at such information. Nearly half of our sample (1,369 subjects, 45.1%) reported talking about the COVID-19 outbreak with family, friends and colleagues for more than two hours per day, 1,116 (36.8%) between one and 2 h, 505 (16.7%) for less than an hour and 44 (1.4%) did not spend time speaking about COVID-19.

Among respondents with children, 451 (26.0%) reported excessive crying or unusual irritation, 308 (17.7%) noticed a behavioral regression, 186 (10.7%) noticed unusual headaches or other unexplained pain sensations, 752 (43.3%) reported anxiety and concern about the COVID-19 outbreak.

Physical activity was reduced in 1,428 (47.0%) subjects in the last 3 weeks, while 125 (6.3%) said they could increase it. The physical contacts diminished in 2,875 subjects (94.9%), increased in 12 (0.4%), and did not change in 143 (4.7%). As opposed to physical contacts, virtual contacts increased for 1,960 individuals (64.6%), diminished for 175 subjects (5.8%), and did not change for 898 (29.6%). Hobbies and other activities undertaken for pleasure or relaxation diminished for 1,908 persons (63%), increased for 260 persons (8.6%), and did not change for 862 (28.4%). A statistically significant correlation was found between an increased number of working hours and stress symptoms such as eating less or more, sleeping less or not feeling relaxed, drinking or smoking more.

One thousand two hundred and eighty-four respondents (42.4%) declared they are separated from their family because of COVID-19.

### Need for Psychological Support and Confidence in the Future

**Figure 2:**
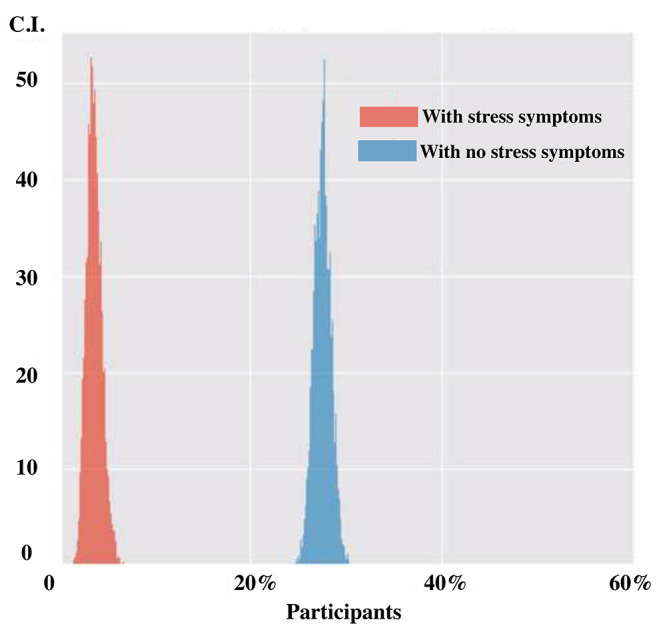
Need for psychological support in participants with and without stress symptoms.

**Figure 3:**
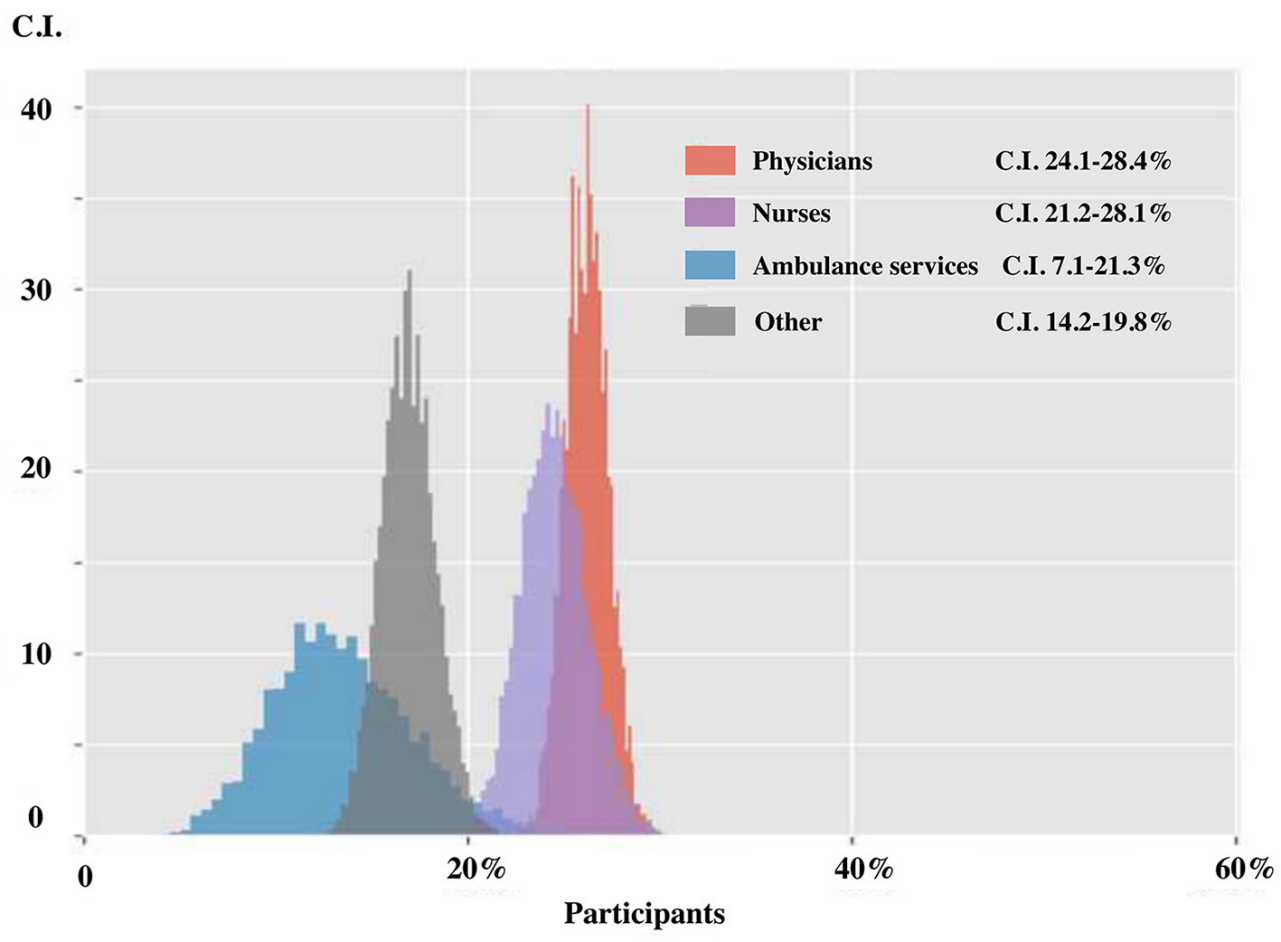
Need for psychological support according to professional categories.

In the last 3 weeks, 706 respondents perceived to need specialized psychological support (23.3%). Confidence in the future was diminished for 1,404 persons (46.3%), increased for 246 subjects (8.1%), and did not change for 1,384 (45.6%). Individuals feeling the need of professional psychological support were more often female (C.I. 24.8–28.3 vs. 13.0–17.1% for females and males, respectively), in a couple (C.I. 23.7–27.4%) or subjects reporting stress symptoms (Figure 2). Long-career workers (C.I. 7.1–15.0%) and ambulance services (C.I. 7.5–20.8%) expressed less often the need for psychological support. As reported in Figure 3, different professional categories expressed different psychological support need. Subjects stating increased confidence in the future were more often males (C.I 5.4–11.6% vs. 9.2–30.2% for females). As expected, among respondents with diminished confidence in the future there was a higher prevalence of females (C.I 34.2–60.6% vs. 32.6–45.7% for males).

### High-Risk Categories for Developing Stress Symptoms Among Healthcare Workers

**Figure 4:**
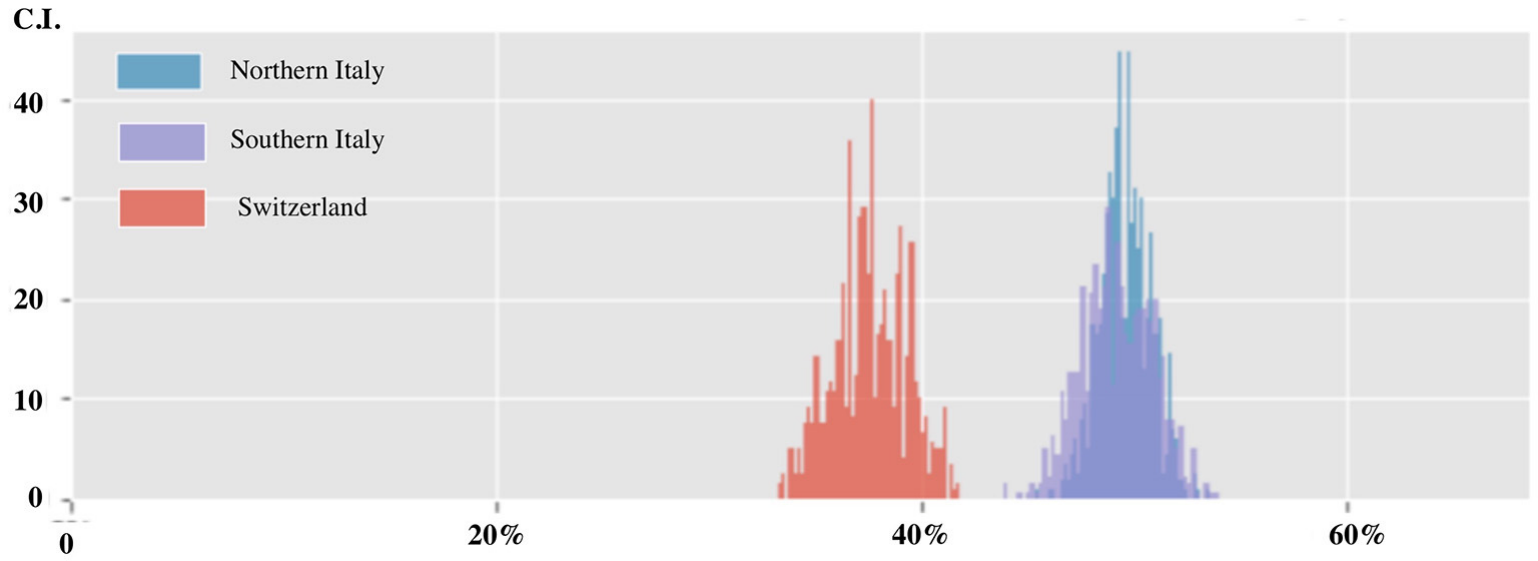
Confidence in the future among healthcare workers divided by geographical region (Nothern Italy, Southern Italy and Swiss).

Plotting the data by country, the confidence in the future in the last three weeks was more lowered in respondents from Italy as compared to Switzerland (C.I. 46.0–52.3% vs. 34.2–41.1%) (see Figure 4). This difference did not change by plotting data either by work category or by age or length of career. Surprisingly, by plotting the decreased confidence in the future for the type of hospital work, all categories were equally affected except of ambulance services (C.I. 16.1–33.6% vs. 40.0–51.2% for other categories).

The decision tree analysis showed a higher probability of sleeping less than normal (accuracy 62.2%) in respondents aged >30 from Italy and having one or more children in their family. In particular, nurses of critical care with a history of anxiety. Finally, history of anxiety resulted to be an independent factor associated to reduced confidence in the future (C.I. 47.7–60.2 vs. 37.6–42.5% with and without a history of anxiety, respectively).

## Discussion

The present study highlights elevated stress levels in a cohort of Italian-speaking hospital workers during the early phases of the outbreak of Sars-Cov2 infection due to the novel COVID-19, independently from the appurtenance to different countries (Italy and Swiss). To the best of our knowledge, our study was the earliest to explore the psychological impact of this novel pandemic on healthcare workers among Italian-speaking countries (Italy and Italian-speaking regions of Switzerland). From our analysis, being female, working as a nurses and subjects having previous problems of anxiety has been the main predictor for referring stress symptoms (Figure 3).

Generally speaking, healthcare workers have been firstly involved in the catastrophic events caused by the massive COVID-19 outbreak (10–12). Beyond demanding clinical and logistic issues, hospital employees have to deal with their own physical and mental health (17–23). Work-related stress has shown to impact physician's mental health, patients' care quality, and the efficiency of the healthcare system (20). Consequences in terms of mental health in the context of maxi-emergency situations may be even more pronounced than normal. Feelings of inadequacy, insufficiency or confusion are common and hospital workers may even experience negative behavioral reactions, depression and illness, possibly leading to lack of effectiveness and efficiency at work (4–6).

The large number of responses and the small percentage of uncompleted surveys (2, 7%) in less than 40 h indicates the high interest in the subject among healthcare personnel. This may also express the need among healthcare workers to communicate, to be heard, and understood that encompasses professional attitude and rules of conduct and touches the psyche and emotions.

More than 50% of survey participants were physicians, likely because the survey promoters were physicians both in southern Switzerland and northern Italy and invitations to participate were sent through personal contacts and social media. However, the high turnout rate of several other hospital categories could mirror the concept that the present is a common burden among all health workers.

### Demographics, Social and Working Distribution

The group aged 26–45 represents the majority of the sample, probably because it is, are more likely to be reached by social media. The small number of respondents aged between 18 and 25 is likely to reflect the relatively low number of very young respondents working in hospitals as confirmed by the fact that medical trainees under 26-years of age are not yet medical doctors in Italy and Switzerland. The distribution of worked years in the hospitals roughly reflects the age distribution.

In our sample, two-thirds of survey participants were female, which could be interpreted to a growing percentage of female medical professionals. In our sample, percentages of married, single, divorced etc., and other demographic characteristics were grossly comparable to the Swiss and Italian population (24, 25).

Nearly one third of hospital workers in our study had to increase their working activity. It represents the first, well-recognized stress factor, proportional to the increase of worked hours (26). As expected, health care professionals having to increase their working hours, are those employed either in critical care settings or general practitioners, the first categories having to be confronted with the COVID-19 emergency. On the other hand, in several hospitals in northern Italy and southern Switzerland, a dramatic reduction of surgical elective cases and outpatient clinic activity has been deemed necessary to contain virus spread and, as a consequence, surgeons as a category experienced a reduction in working hours. Notwithstanding, a reduction of working hours should be considered insidious, as it could nonetheless jeopardize the mental status and could lead to depressive symptoms, hopelessness, and uselessness (27).

### Stress Symptoms

Sleep disturbances can be caused by stress and be related to Post-Traumatic Stress Disorder (PTSD) and the first response is generally considered a period of arousal and wakefulness (28, 29). A great proportion of hospital workers in our study declared to sleep less than normal and to feel less restored by sleep in general, presumably as a reaction to the stressful circumstances and it is likely to reflect the high prevalence of sleep arousal and anxiety symptoms in healthcare professionals.

The correlation analysis showed a relatively higher prevalence of stress symptoms among young professionals with at least one child to care for, as a response of having to face the COVID-19 outburst emergency.

Similarly, an increase or a decrease in in food intake may mirror a reaction to a stressful situation and, actually, in our sample, only 40% of the interviewed reported no change (30). Moreover, among smokers, there was a high proportion of participants that declared to have increased the number of cigarettes per day in the first 3 weeks since the outbreak onset. A smaller, but significant proportion of respondents reported an increase in alcohol consumption. All these behavioral changes can be considered stress symptoms (31, 32). In our study, a strong correlation was found between the above-mentioned symptoms and the need for psychological support. Respondents reporting such symptoms were more prone to report the need for psychological support. As far as we know, this is the first time that such a need is quantified within the emergency of COVID-19.

### Need for Psychological Support

Interestingly, respondents with children reported the most stressful answers and felt the need for professional psychological support more often than their non-parenting peers (accuracy 62.2%). Despite some might consider this obvious, such data are nonetheless of outstanding importance, as hospital employees are often in the fertile age range. In the setting of a massive viral outbreak, hospital caregivers experience important issues in caring for their children because of the closure of schools and other facilities. Parents probably do not experience only the stress related to future uncertainty but also the fear of getting infected and possibly transmit such an infectious disease to their offspring. Specifically, COVID-19 has shown to be particularly infective also for hospital workers and casualties have been reported among hospital staff (10–12). Coherently, many hospital workers with children (nearly 95%) declared to have reduced physical contact with other family components.

Almost all subcategories of hospital workers perceive the stress related to the outbreak equally. Only long-career workers and ambulance service personnel reported needing psychological support less as compared to other categories. This may be explained by the high level of experience and long-standing training in stress management in long-career employees. On the other hand, emergency services personnel may be used to address stressful situations, as they are part of a coordinated and ordered emergency response and have to constantly handle very high levels of stress (33, 34).

The actual outbreak could be a repeated trauma for many healthcare categories, putting them at risk of psychiatric sequelae such as PTSD (8, 35). It is of outstanding importance in such a critical situation to promptly implement measures to mitigate the impact of the emotional burden of the present COVID-19 pandemic while at the same time dealing with its clinical challenges (2, 4).

Positive behaviors such as healthy eating, sport practicing, and sleeping an adequate number of hours have shown to impact and reduce the impact of stress (36). Many other strategies have also shown to be effective, such as the implementation of debriefing sessions and group therapies to share experiences and relieve the sorrow related to challenging and stressful situations. Programs of de-escalation of tension through mindfulness techniques could be also cost-effective and easily implemented in routine practice to prevent future development of acute and chronic PTSD, major depression or suicidal behaviors (37–39).

Our study has several limitations. Firstly, our data were collected in a completely anonymized and we have not certainty on their truthfulness. Moreover, a biased sample of subjects' more at risk of developing stress symptoms could have been more induced to answer the survey or, on the contrary, maybe busiest, or more stressed healthcare workers did not access social media. In addition, another limitation that has to be mentioned is the fact that we did not have enough data for discriminating stress symptoms among healthcare workers that were asked to do professional tasks out of their area of expertise.

Furthermore, the CwCOVID-19 questionnaire had not been previously validated. This may limit the comparisons with other studies but enables the evaluation of pandemic-related stress symptoms. This approach was inspired by similar studies during other pandemics (9) or the present (10).

Nonetheless, our data denote a certain internal coherence, that can be interpreted as trustworthiness. Here we present the very first evaluation on stress symptoms in a representative sample of healthcare professionals in the European continent, that was composed of (mainly) medical doctors but also of several types of healthcare workers.

## Conclusions

Perceived psychological burden among healthcare workers should be considered relevant since the early phases of an infectious disease widespread. COVID-19 outbreak has, therefore, to be considered as a major stressor, that was able, since the beginning, to threaten the physiological and psychological integrity of healthcare professionals. This may lead hospital efficiency at jeopardization. Facing the following events of 2020 and the still ongoing 2021, healthcare workers should be under close psychological monitoring and long-term psychological counseling for them should be provided.

Further studies in different time points of the outbreak are needed to understand the impact of COVID-19 emergency on healthcare workers' psychology and mental health.

## Author's Note

LU is an Emergency Medicine specialist (MD, PhD) who received a 4-year-special-training in Cognitive Behavioral Therapy at the Cognitive Behavioral Therapy School of Milan (https://studicognitivi.it). SaU (MD) is a psychiatrist and neurologist trained in developmental medicine. All the authors have scientifically and clinically shared working experience, have published past researches on impacted journals and are committed to ameliorate healthcare workers job conditions.

## Data Availability Statement

The original contributions presented in the study are included in the article/Supplementary Material, further inquiries can be directed to the corresponding author/s.

## Ethics Statement

Ethical review and approval was not required for the study on human participants in accordance with the local legislation and institutional requirements. The patients/participants provided their written informed consent to participate in this study.

## Author Contributions

LU, SaU, and LP designed the study. LU was involved in constructing the dataset, interpreting data, and drafting the manuscript. PM-H, FM, StU, CZ, and LP helped in data collection and revising the final questionnaire. All authors discussed the results and commented on the manuscript.

## Conflict of Interest

The authors declare that the research was conducted in the absence of any commercial or financial relationships that could be construed as a potential conflict of interest.

## Publisher's Note

All claims expressed in this article are solely those of the authors and do not necessarily represent those of their affiliated organizations, or those of the publisher, the editors and the reviewers. Any product that may be evaluated in this article, or claim that may be made by its manufacturer, is not guaranteed or endorsed by the publisher.
