# Supplementary material for: Psychological Impact of the Very Early Beginning of the COVID-19 Outbreak in Healthcare Workers: A Bayesian Study on the Italian and Swiss Perspectives
Source: Front Public Health. 2022 Mar 24;10:768036. doi: 10.3389/fpubh.2022.768036 (PMC8987285; doi:10.3389/fpubh.2022.768036)
Supplement: Supplementary file 1 [file Data_Sheet_1.docx]

Supplementary Material

**Coping with CoVid-19 Questionnaire**

We ask you to answer this short questionnaire. It will take you less than 5 minutes and you will help to give an idea about the hospital workers’ condition during this emergency situation.

**1. Age?**

18-25 26-35 36-45 46-55 56-65 >65

**2. Gender?**

Male Female

**3. Civil status?**

Married Cohabiting Civil Union Divorced Single Widow

**4. How many people live in your family?**

1 2 3 4 5 > 5

**5. Are there children or people < 18-year-old in your family?**

Yes No

**6. How many people > 65-year-old live in your family?**

1 2 3 More than 3 None

**7. How many years have you been working in hospital?**

0-1 y 2-5 y 5-10 y 10-20 y 20-30 y > 30 y

**8. In which region do you work?**

_________________

**9. Which kind of job do you do in hospital?**

Nurse Obstetrician Ambulance Physician Technician Pharmacy Canteen

Administrative Other

**10. In which service do you work?**

Ambulance General practitioner Internal medicina Pediatry Geriatry Neurology

Surgery Emergency Department Cardiac Surgery Gynecology Urology

Neurosurgery Ear/Nose/Throath Intensive care unit Psychiatry Oncology

Rehabilitation Technical service Pharmacy Radiology Anesthesia

Orthopedy Cardiology Other

**11. Did you have to work more than normal due to the CoVid-19 emergency?**

Yes, up to 10 hours per week Yes, up to 20 hours per week

Yes, up to 30 hours per week Yes, >30 hours per week No

**12. Did you have to reduce your work due to the CoVid-19 emergency?**

Yes, up to 10 hours per week Yes, up to 20 hours per week No

Yes, >20 hours per week I was quarantined I was CoVid +

**13. Did your sleep change in the last 3 weeks?**

I sleep less I sleep more I sleep the same but I feel less rested

I sleep the same but I feel more rested No change

**14. Did your eating habit change in the last 3 weeks?**

Yes, I eat more Yes, I eat less I eat the same quantity but healthier food No change

I eat the same quantity but more unhealthy food

**15. Did you notice a worsening of your chronic health disease in the last 3 weeks?**

Yes No I have no chronic health disease

**16. Did you notice new or unusual symptoms in the last 3 weeks?**

Yes, pulmonary symptoms Yes, palpitations Yes, pain Yes, other No new symptoms

**17. Do you smoke?**

Yes, I started in the last 3 weeks Yes, I started before the last 3 weeks

No, I quitted in the last 3 weeks No, I quitted before the last 3 weeks I never smoked

**18. Did you recently increase the cigarettes/cigars/e-cigarettes number per day?**

Yes No I don’t smoke

**19. Do you drink alcohol?**

Yes, I started in the last 3 weeks Yes, I started before the last 3 weeks

No, I quitted in the last 3 weeks No, I quitted before the last 3 weeks No, I don’t drink

**20. Did you recently increase the alcohol consumption?**

Yes No I don’t drink

**21. Do you use drugs?**

Yes, I started in the last 3 weeks Yes, I started before the last 3 weeks

No, I quitted in the last 3 weeks No, I quitted before the last 3 weeks No, never

**22. Did you recently increase drugs consumption?**

Yes No No, never used

**23. How much time do you spend every day on getting information about the CoVid-19 outbreak (except due to working reasons)?**

Less than 1 hour 1-2 hours >2 hours I don’t look at such information

**24. How much time do you spend every day on speaking about CoVid-19 with friends, relatives, colleagues?**

Less than 1 hour 1-2 hours >2 hours I don’t speak about such information

**25. Are you divided from your family due to the CoVid-19?**

Yes No

**26. Did you notice in your children excessive crying or irritability in the last 3 weeks?**

Yes No I have no children

**27. Did you notice a regression to unappropriated behaviors for the age of your children in the last 3 weeks?**

Yes No I have no children

**28. Did you notice in your children headache or unexplained pain?**

Yes No I have no children

**29. Did you notice in your children anxiety or concerns due to the CoVid-19?**

Yes No I have no children

**30. Dou you practice regular physical activity?**

Yes, once a day Yes, 4-5 per week Yes, 2-3 per week Yes, once a week No

Yes, less than once a week

**31. In the last 3 weeks, your physical activity…**

Increased Decreased Did not change I don’t do physical activity

**32. Your physical contacts… in the last 3 weeks**

Increased Decreased Did not change

**33. Your virtual contacts… in the last 3 weeks**

Increased Decreased Did not change

**34. Your favorite activities, your hobbies in the last 3 weeks are…**

Increased Decreased Did not change

**35. In the last 3 weeks, did you feel the need for a qualified psychological support?**

Yes No

**36. Did you suffer in the past from anxiety?**

Yes No I don’t know

**37. Did you use in the past medications to control your anxiety?**

Yes No

**38. In the last 3 weeks, your confidence in the future is…**

Increased Decreased Did not change
